# Intraoperative rupture of intracerebral aneurysm immediately after meningioma resection: a case report

**DOI:** 10.1186/s12883-022-02664-8

**Published:** 2022-04-11

**Authors:** Shingo Tanaka, Masaaki Kobayashi, Toshiya Ichinose, Nozomu Oikawa, Masashi Kinoshita, Akifumi Yoshikawa, Naoyuki Uchiyama, Mitsutoshi Nakada

**Affiliations:** 1 Department of Neurosurgery, Division of Neuroscience, Graduate School of Medical Science, Kanazawa University, 13-1 Takara-machi, Kanazawa, Ishikawa 920-8641 Japan; 2 Department of Neurosurgery, Ishikawa Prefectural Central Hospital, Ishikawa, Japan

**Keywords:** meningioma, Brain tumor co-existing with cerebral aneurysm, Feeder related aneurysm, Intraoperative ruptured aneurysm

## Abstract

**Background:**

Meningiomas and unruptured cerebral aneurysms (UCAs) rarely coexist. However, the treatment strategy remains to be fully elucidated. This report is a first report that UCA related to the tumor feeder intraoperatively ruptured when the meningioma was resected.

**Case presentation:**

Herein, we present a case of meningioma coexisting with contralateral UCA related to a tumor feeder. Immediately after the meningioma was resected, intraoperative acute brain swelling due to rupture of the contralateral aneurysm appeared. The swollen brain protruding into the epidural space was resected, following contralateral ruptured aneurysm was performed by endovascular surgery. Intensive neurological treatment was administered and the patient gradually recovered.

**Conclusion:**

This report highlights the possibility of intraoperative UCA rupture related to the tumor feeder when the meningioma is resected.

**Supplementary Information:**

The online version contains supplementary material available at 10.1186/s12883-022-02664-8.

## Background

The co-existence of a primary brain tumor and an unruptured cerebral aneurysm (UCA) is rare. The incidence has been reported to be 0.5 − 4.5% [1, 2]. Among brain tumors coexisting with UCA, meningioma accounts for the highest rate (29.3 − 60%) [1, 2]. Statistical studies of meningioma coexisting with UCA have reported a frequency of 1.1 − 7.7% [3–6]. The treatment guidelines for meningioma coexisting UCAs have not been established, and the treatment priority is controversial [4, 5, 7–9]. We encountered a case of right frontal convexity meningioma coexisting with left UCA related to a tumor feeder. To our knowledge, this is the first case that coexisting contralateral UCA at the tumor feeder ruptured immediately after meningioma resection during surgery.

## Case presentation

**Fig. 1:**
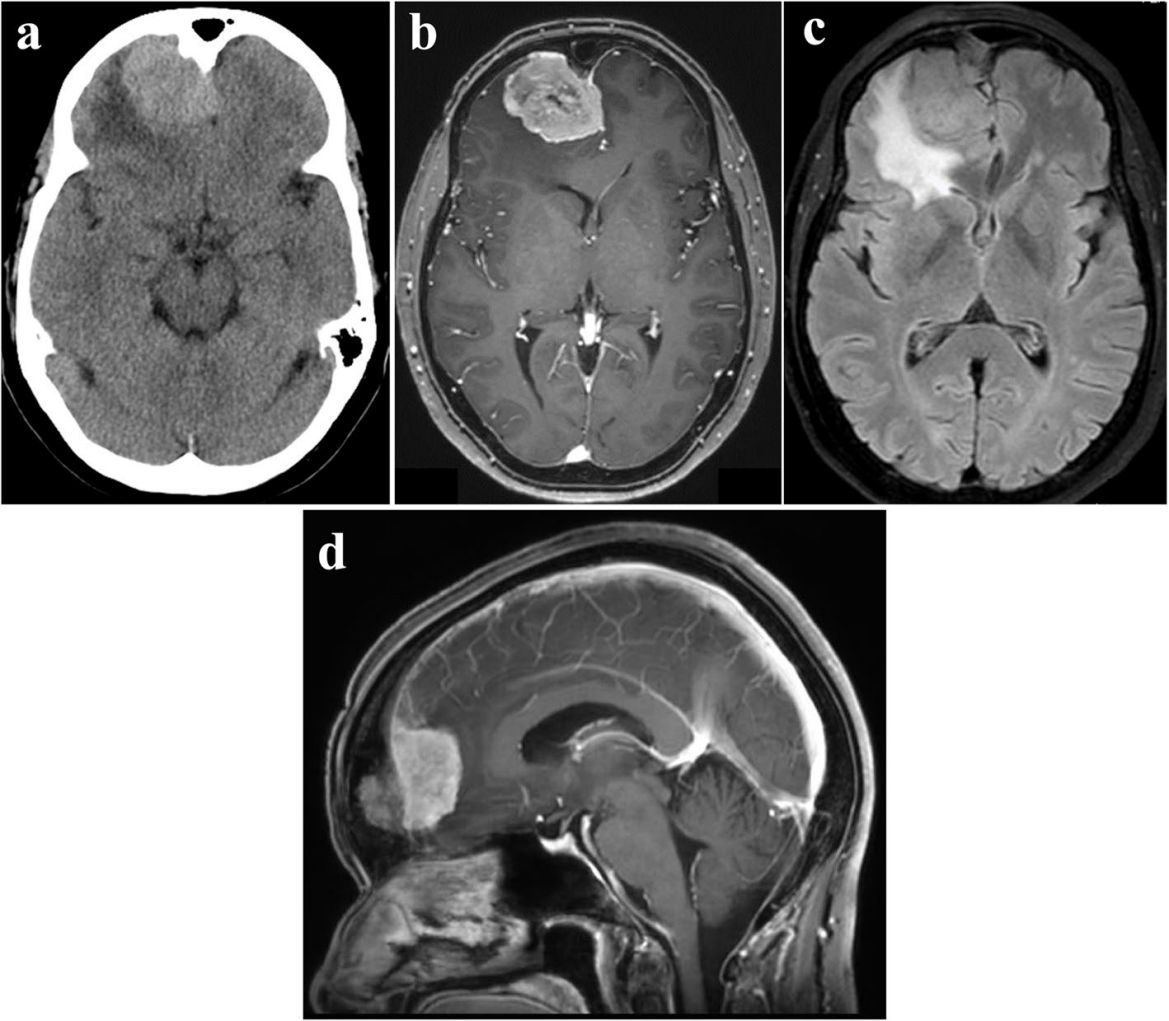
Preoperative radiographic imaging. **a** Axial view of CT showing an isodensity tumor lesion in right frontal region. **b** Gadolinium (Gd)-enhanced T1-weighted image shows homogenous enhanced tumor. **c** Fluid-attenuated inversion-recovery (FLAIR) image demonstrates brain edema surrounding tumor. **d** Sagittal view of Gd-enhanced T1-weighted image shows that the bottom of tumor does not reach the anterior skull base

**Fig. 2:**
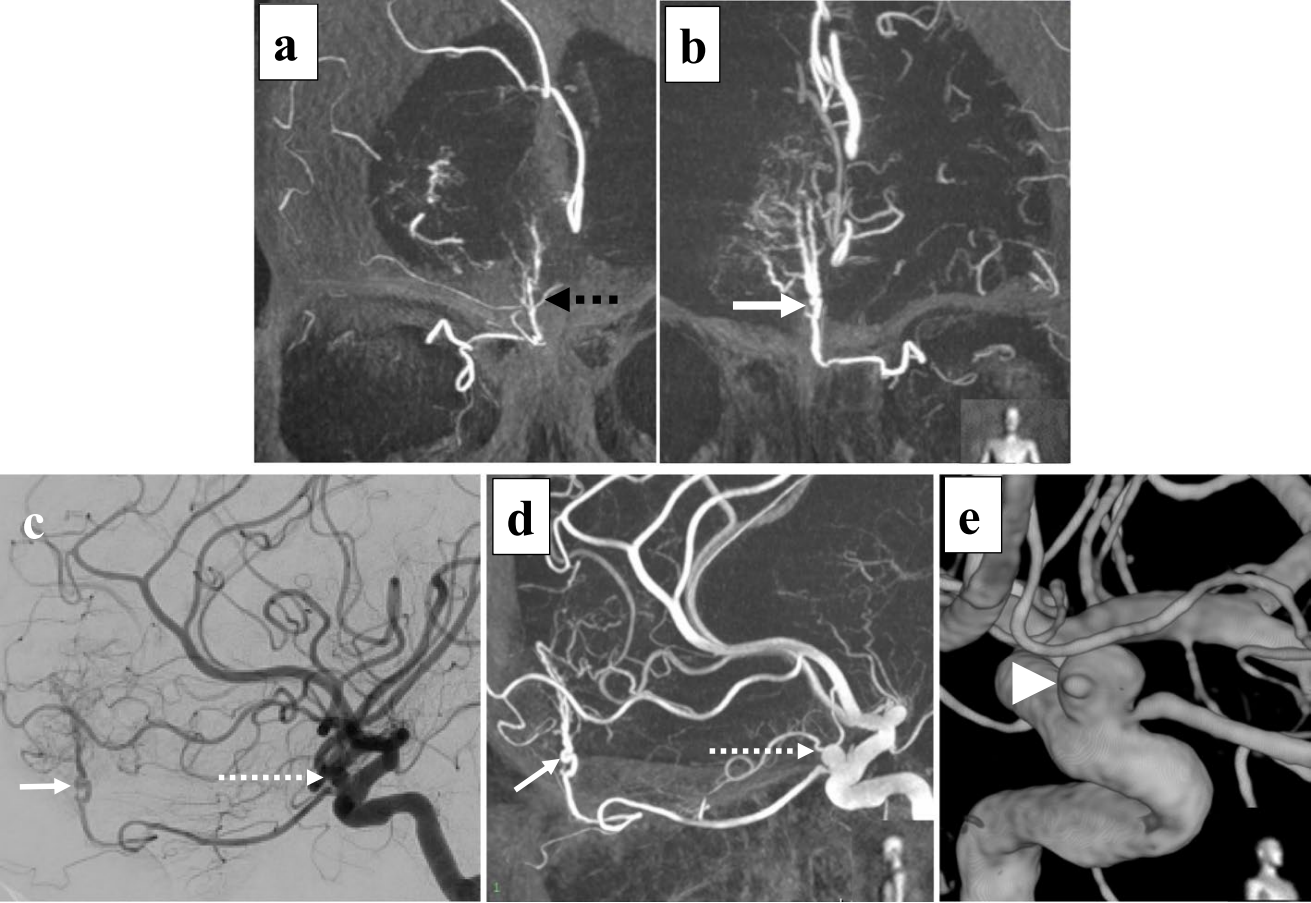
Digital subtraction angiography (DSA) of internal carotid artery (ICA). **a** Coronal view of Maximum Intensity Projection (MIP) image shows ipsilateral, right tumor feeder (black dotted arrow). **b** MIP image shows contralateral, left tumor feeding artery (white arrow). **c** Lateral view of DSA of left ICA shows left tumor feeder (white arrow) arose from left ophthalmic artery (OphA) and left ICA-OphA aneurysm (dotted line arrow). **d** Lateral view of MIP image shows that left feeding artery (white arrow) and ICA-OphA aneurysm (dotted line arrow). **e** 3D-reconstruction view demonstrates a bleb formation (white arrowhead) in ICA-OphA aneurysm wall

A 52-years-old female was referred to our hospital because of a persistent morning headache. Computed tomography (CT) revealed a right frontal lobe tumor measuring approximately 4.1 × 3.1 cm with bony hyperostosis (Fig. 1a). Magnetic resonance imaging (MRI) revealed that the tumor was enhanced by gadolinium and was accompanied by a dural tail sign and strong peritumoral edema (Fig. 1b, c). She was diagnosed with a right frontal convexity meningioma. Digital subtraction angiography (DSA) demonstrated that the main feeding vessels of the tumor were the bilateral ethmoidal arteries arising from the ophthalmic artery (OphA). The vascularization of the meningioma was more significant in the left-sided feeding artery than in the right-sided feeding artery (Fig. 2a, b). Additionally, a left internal carotid artery-ophthalmic artery (ICA-OphA) aneurysm with a bleb was observed (Fig. 2c, d, e). Its maximum diameter was 5.5 mm. The aneurysm was not in contact with the tumor (Fig. 1d, 2c, 2d).

**Fig. 3:**
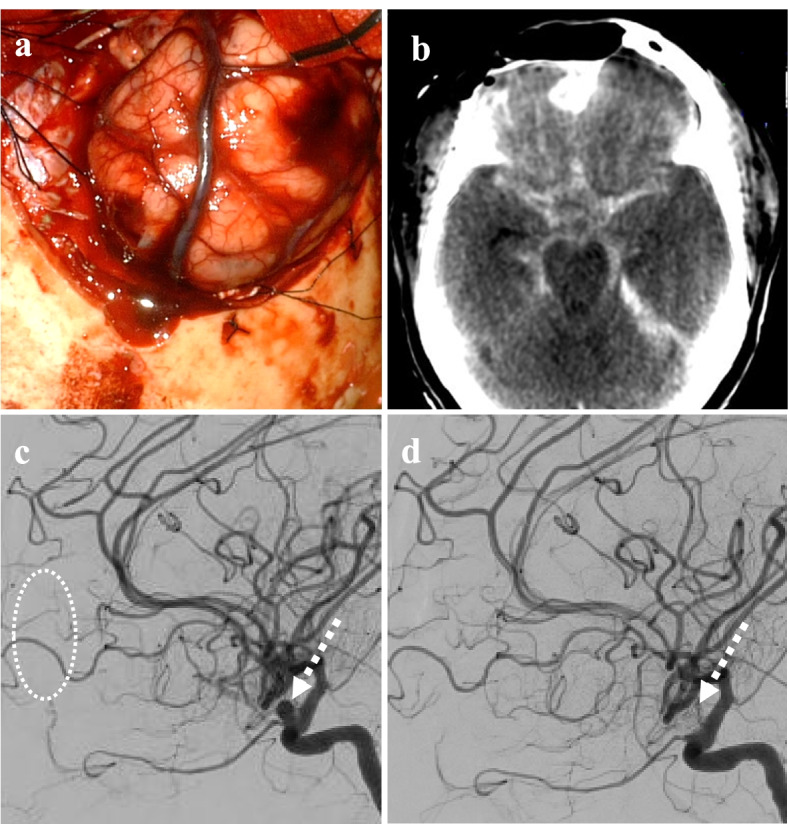
Intraoperative event immediately after tumor resection showing. **a** The photograph shows acute brain swelling after meningioma removal. **b** CT identified subarachnoid hemorrhage. **c** and (**d**) Lateral view of left ICA of DSA demonstrates disappearance of tumor feeder (dotted white circle) and complete coil-embolization for ICA-OphA aneurysm (dotted line arrow)

Surgery for symptomatic brain tumor was performed by right frontal craniotomy (7.5 × 5.0 cm). The intraoperative frozen section diagnosis was a meningioma without obvious malignancy. The interface between the tumor and the surrounding brain was tightly adhered. The tumor interface was created in the following order: posteriorly, laterally, and anteriorly under a microscope. The medial border of the tumor is the surface of the falx. Finally, the bottom surface of the tumor was detached after coagulation of the feeding artery. Immediately after the en bloc resection of the tumor, the right frontal lobe appeared to swell rapidly (Fig. 3a). Additional craniotomy of the right parietal and left frontal regions and resection of the swollen brain protruding into the epidural space was performed (see additional file 1). The wound was closed without returning the skull bone flap, and she underwent intracranial scrutiny under anesthesia. Subarachnoid hemorrhage (SAH) was observed on CT (Fig. 3b). The patient was promptly transported to the angiography room. DSA findings showed that the tumor feeder that arose from the left OphA disappeared, and the left ICA-OphA aneurysm was recognized preoperatively (Fig. 3c). An examination of the cause of SAH revealed no findings other than the left ICA-OphA aneurysm, which could cause bleeding. Endovascular surgery for this ruptured aneurysm was performed with systemic heparinization to prevent acute thrombosis. A 6-Fr shuttle guiding sheath (Cook Medical, Bloomington, IN, USA) was introduced into the right ICA from the right femoral artery. A microcatheter, SL-10 STR (Stryker, Kalamazoo, MI, USA), was inserted into the aneurysm. Coil embolization was performed using the balloon-assisted technique with Scepter XC (Terumo, Tokyo, Japan) (Fig. 3d). The time required for endovascular treatment was 1.5 h. Subsequently, the patient returned to the operating room for right extra-ventricular drainage and skin closure. Immediately after the operation, thiopental (4 mg/kg/h) was continuously administered for 72 h to control intracranial pressure. Fasudil hydrochloride (90 mg/day) and ozagrel sodium (80 mg/day) combination therapy was administered for 14 days to prevent cerebral vasospasm-related SAH from day 3 post-surgery. Her impaired consciousness tended to recover. On day 37 post-surgery the patient’s consciousness level fully recovered. The histological diagnosis was transitional meningioma (WHO grade I). Follow-up MRI showed a total resection of the meningioma. She underwent rehabilitation and was discharged home approximately 3 months after surgery. Cognitive assessment was not performed during rehabilitation. Functional outcome at discharge was assessed using the Functional Independence Measure (FIM™) [10]. FIM™ motor and cognitive score were 85 and 26 points, respectively, suggesting that her Activities of Daily Living (ADL) was relatively well maintained. She had gradually recovered by 6 months post-surgery without any neurological deficit.

## Discussion and conclusions

The treatment strategy for meningioma coexisting with UCA has not been established and is controversial [2, 4, 5, 7–9]. The consensus is that lesions with symptoms might be prioritized for treatment [4–6]. UCA co-existence of meningiomas can be divided into three categories: UCAs unrelated to tumor feeders, UCAs encased in or near to the meningioma, and UCAs related to tumor feeders [2, 6, 8, 11–15] such as in our case. There are no reports on the rupture rate of UCA after meningioma resection; however, there are reports regarding treatment of UCAs followed by meningioma resection or UCAs and meningioma treated at the same time [4, 5, 9, 15]. This indicates that treatment of UCAs might not be considered essential because there are cases in which UCAs disappeared after meningioma removal [12] and no report of ruptured UCAs in follow-up after meningioma removal. In this case, the reasons for prioritizing meningioma removal are as follows: 1) the meningioma was symptomatic, without evidence of SAH; 2) the 3-year rupture rate or 5-year rupture rate of UCA in the same area based on a prospective study was as low as 0.6% and 0.4%, respectively [16, 17]; 3) the right frontal meningioma was clearly apart from left ICA-OphA aneurysm. Therefore, this case was not in a high risk group such as a case that intracranial aneurysm was encased by meningioma or was in contact with the meningioma [4].

In this case, the contralateral ICA-OphA aneurysm ruptured immediately after removal of the meningioma. This aneurysm with a bleb may rupture coincidentally. However, the intraoperative rupture of this aneurysm could be associated with meningioma removal because this ICA-OphA aneurysm was categorized as a UCA related to the tumor feeder. Although there are no reports on the mechanism of ruptured UCA-related tumors, hemodynamic factors may be the cause of UCA rupture [18]. Changes in blood flow might be induced by meningioma removal, and hemodynamic stress might contribute to the intraoperative rupture of the contralateral ICA-OphA aneurysm. Hypertension that she is on medical treatment might be related the aneurysm rupture. Aggressive hypotensive anesthesia may have prevented intraoperative rupture of the intracranial aneurysm although systolic blood pressure was maintained at 100–120 mmHg during operation.

This case showed intraoperative acute brain swelling due to SAH with a ruptured aneurysm after tumor resection. Surgical options for reducing intracranial pressure include decompressive craniectomy or lobectomy/parenchymal resection [19–21]. In this case, an additional craniectomy was performed on the ipsilateral and contralateral sides. The treatment of ruptured aneurysms involves clipping or endovascular coiling. In this case, endovascular coiling was performed for the ruptured aneurysm. The reason for coiling selection was as follows: 1) the dome-to-neck ratio of the aneurysm was 1.7, which can be treated by endovascular coiling [22]; 2) contralateral frontal lobe damage should be avoided to prevent the exacerbation of cognitive function.

In conclusion, the possibility of rupture co-existing UCAs associated with tumor feeders after tumor removal might be kept in mind.

## Supplementary Information

**Additional file 1**. Shows the operative video.

## Data Availability

The datasets used and/or analyzed during the current study are available from the corresponding author on reasonable request.

## Abbreviations

UCA: Unruptured cerebral aneurysm
ICA: Internal carotid artery
OphA: Opthalmic artery
SAH: Subarachnoid hemorrhage
WHO: World Health Organization
FIM™: Functional Independence Measure
ADL: Activities of Daily Living
